# Brain-Inspired Architecture for Spiking Neural Networks

**DOI:** 10.3390/biomimetics9100646

**Published:** 2024-10-21

**Authors:** Fengzhen Tang, Junhuai Zhang, Chi Zhang, Lianqing Liu

**Affiliations:** 1 State Key Laboratory of Robotics, Shenyang Institute of Automation, Chinese Academy of Sciences, Nanta Street 114, Shenyang 110016, China; zhangjunhuai24@mails.ucas.ac.cn (J.Z.); zhangchi@sia.cn (C.Z.); lqliu@sia.cn (L.L.); 2 School of Computer Sciences, University of Chinese Academy of Sciences, Beijing 100049, China

**Keywords:** spiking neural networks, self-adaptive coding, surrogate gradient backpropagation, leaky integrate-and-fire neuron model

## Abstract

Spiking neural networks (SNNs), using action potentials (spikes) to represent and transmit information, are more biologically plausible than traditional artificial neural networks. However, most of the existing SNNs require a separate preprocessing step to convert the real-valued input into spikes that are then input to the network for processing. The dissected spike-coding process may result in information loss, leading to degenerated performance. However, the biological neuron system does not perform a separate preprocessing step. Moreover, the nervous system may not have a single pathway with which to respond and process external stimuli but allows multiple circuits to perceive the same stimulus. Inspired by these advantageous aspects of the biological neural system, we propose a self-adaptive encoding spike neural network with parallel architecture. The proposed network integrates the input-encoding process into the spiking neural network architecture via convolutional operations such that the network can accept the real-valued input and automatically transform it into spikes for further processing. Meanwhile, the proposed network contains two identical parallel branches, inspired by the biological nervous system that processes information in both serial and parallel. The experimental results on multiple image classification tasks reveal that the proposed network can obtain competitive performance, suggesting the effectiveness of the proposed architecture.

## 1. Introduction

The human brain is a very powerful yet complex computational system. It contains approximately 90 billion neurons interconnected by trillions of synapses [1,2]. Inspired by the nervous system, artificial neural networks (ANNs) have been successfully used in various application areas, such as speech recognition, visual object recognition, and target detection [3,4]. However, ANNs only mimic the biological nervous system abstractly and roughly, ignoring its complex temporal dynamics [1]. To overcome this limitation, spiking neural networks (SNNs) have emerged, which are more biologically plausible. Unlike traditional ANNs, the discrete action potentials or spikes are used in SNNs to encode and transmit information [5,6]. The precise timing of spikes may also be used to encode information in biological neural networks [7]. Thus, communication using spike trains in SNNs can convey much richer information than that used in the firing rates commonly used in traditional ANNs. Thanks to their high resemblance to biological nervous systems, study on SNNs may help to better understand the mechanism of the brain. Furthermore, the ability to capture the temporal dynamics of biological neurons enables SNNs to process spatiotemporal information through spike learning and memory mechanisms [8,9]. Moreover, SNNs are efficient and energy-conservative because their neurons are excited only when they receive input spikes. Therefore, SNNs have recently emerged as a new computing paradigm, showing good prospects for many real-world applications [10].

Currently, many works have been devoted to SNNs. Leaky integrate-and-fire (LIF) models are commonly used in SNNs to model neuron dynamics [1]. A two-layer SNN with lateral inhibition and an adaptive spiking threshold is proposed in [11]. The proposed network is trained by adopting a biologically plausible learning rule referred to as spike-timing-dependent plasticity (STDP) [7], which is an unsupervised learning algorithm. Simply modifying the connection weights by STDP may lead to poor results [12]. To alleviate this problem, the authors of [12] extend the unsupervised STDP learning rule to a supervised one, obtaining improved performance. The authors of [13] describe a feedforward spiking neural network based on temporal coding where the input-output relation is differentiable almost everywhere such that the backpropagation (BP) training methods for ANNs can directly be applied. Along this line, the continuous LIF model was transformed into an iterative updating version, and a surrogate derivative was introduced for the nondifferentiable spiking activity function; subsequently, a backpropagation (BP) algorithm was successfully applied to train deep SNNs [14]. The successful application of the BP algorithm and its variants to training deep SNN models [15] has led to better results of SNNs in learning tasks, such as speech recognition and image classification.

To further improve their performance, SNNs are combined with convolutional neural networks (CNNs). The authors of [16] propose a CNN-SNN (CSNN) model that combines SNN with CNN in a rather loose way. It stacks the fully connected layer of the SNN on top of a convolutional and pooling layer in the CNN, achieving comparable performance to other cognitive models with fewer neurons and training samples. In contrast, the authors of [17] used spiking neurons with convolutional receptive fields to build the deep SNN network, which is directly trained by the spatiotemporal backpropagation (STBP) algorithm, achieving better recognition results on image datasets. In [18], a spiking deep convolutional neural network is proposed that uses a difference-of-Gaussian (DOG) filter in the first convolutional layer to extract edge information and then encodes the pixel values of the input image as delayed spikes through the DOG filter. The authors of [19] proposed an auto-encoder model to train the spiking deep convolutional neural network in an unsupervised manner. The authors of [20] suggest using lateral interactions to improve spiking convolutional neural networks. The lateral interactions between neighboring neurons are modeled and included in the update formula of the spiking neuron membrane potential.

Most of the existing SNNs require a separate spike-encoding preprocessing step to convert the real-valued inputs into discrete spikes first and then input the spikes to the network for further processing [21]. The spike-encoding process is dissected from the spiking neural network and is not trained together with the subsequent network. There exist two methods to convert real-valued inputs into spikes, namely rate coding and temporal coding. Temporal coding uses the relative timing of individual spikes to encode information [22,23]. More models adopt rate coding, which uses the frequency of the spike train within a time window to encode information [24,25]. The most commonly used rate encoding is Poisson encoding [17]. The real-valued images are converted into spike trains with firing rates proportional to the pixel intensities. The spikes are sampled according to the Poisson distribution, for which the mean and variance values equal the firing rate. Thus, a long simulation time is required in order to reduce information loss during the input spike encoding, which, instead, increases the computational time of the SNN. Nevertheless, the dissected spike-coding process may inevitably cause information loss in terms of the inputs, which may lead to degenerated performance [26].

However, the biological neuron system does not perform a separate preprocessing step. Instead, the neural pathway directly admits the input from the environment via the sensory receptors to convert analog stimuli into corresponding discrete spikes and subsequently relay information to the central nervous system for further processing. The sensory receptors are not independent of the central neural system but are modulated by the central neural system. To mimic this integrated neural pathway, we incorporate the input-encoding process into the spiking neural network architecture via convolutional operations.

Moreover, the study of the biological brain has revealed that the nervous system does not have a single pathway with which to respond to and process external stimuli; it allows multiple circuits to perceive the same stimulus [27]. For instance, visual stimuli (i.e., the shape or color of an object) can be processed by either the left or right eyes. The visual information from the two eyes converges in cortical neurons for the same perception of the visual stimuli [28]. This redundant configuration facilitates the robustness of the nervous system [29,30]. The impairment of one pathway will not induce catastrophic loss in perception. Therefore, a parallel architecture of an SNN may lead to better performance with high robustness [31]. In this paper, on top of the integrated adaptive input spike-encoding scheme, we propose a parallel SNN architecture, hopefully achieving improved generalization performance and enhanced robustness.

The main contributions of this paper can be summarized as follows:

- Inspired by the integrated neural pathway that directly accepts input from the environment via sensory receptors, we propose a self-adaptive coding spiking neural network (SCSNN) that incorporates the input spike-encoding process into the SNN network via convolutional operations such that the SNN can directly admit real-valued input. The basic framework consists of convolutional layers, pooling layers, and fully connected layers. The convolutional layers use the integrate-and-fire (IF) neuron model to realize the interaction between layers over time throughout the network. The fully connected layer uses the leaky integrate-and-fire (LIF) neuron model to allow the whole network to accumulate over time between layers to accomplish specific classification tasks.
- Motivated by the finding that the biological nervous system may allow multiple pathways to process the same external stimuli in parallel, leading to improved robustness, we construct a parallel SCSNN. Two architecturally identical subnetworks with different parameter initialization are designed. The final prediction of the network is the average output of its subnetworks.
- A backpropagation algorithm is utilized to train the proposed network, where the derivative of an arctangent or sigmoid function is used to approximate the derivative of spike activity.

The rest of this paper is organized as follows. In Section 2, we provide a detailed description of the proposed method. The experimental results are given in Section 3. The main findings and conclusions are presented in Section 4.

## 2. Methods

In this section, we describe the proposed brain-inspired architecture for the spiking neural network in detail.

### 2.1. Spiking Neuron Models

A neuron releases spikes only when its membrane potential exceeds a threshold. Several spiking neural models have been proposed to model this characteristic. In this paper, a modified integrate-and-fire (IF) neuron model and a leaky integrate-and-fire (LIF) [32] neuron model are used.

The IF neuron model can be seen as an ideal integrator, where the voltage of the IF neuron will not decay. The subthreshold neural dynamics are described by a differential equation, as in [33]:

(1) $$\begin{array}{ccc} \frac{dV(t)}{dt} & = & \frac{1}{C}I\left(t\right) \end{array}$$

where V(t) is the membrane potential of the neuron, I(t) is the input current, and *C* represents the capacitance, which is a constant. By discretizing the ordinary differential equation of the IF model, we obtain

(2) V(t)=V(t−1)+X(t)

Here, V(t−1) represents the membrane potential at the previous time, and X(t) denotes the external input ($X\left(t\right)=\frac{1}{C}I\left(t\right)$). The membrane potential of the IF neuron is driven by an external input current, and voltage accumulates until the neuron is fired. Once it is fired, the accumulation of the input current starts from scratch. The state function specifying whether a spike is discharged is given as follows:

(3) $$\begin{array}{c} S\left(V\left(t\right)\right)=\left\{\begin{array}{ccc} 1, & & V\left(t\right)\geq V_{th}\\ 0, & & otherwise \end{array}\right. \end{array}$$

where $V_{th}$ is a threshold. If V(t) is greater than the threshold $V_{th}$, the neuron discharges a spike. $V_{rest}$ is the resting potential of the membrane and is set to 0 in this paper. When a spike is fired, as in the previous time step, the membrane potential resets to the resting potential, and the voltage accumulates from scratch, i.e., $V\left(t\right)=V_{rest}+X\left(t\right)$; when a spike is not fired, the membrane potential of the neuron continues to accumulate from the previous time step, i.e., V(t)=V(t−1)+X(t).

The LIF neuron model is proposed, based on the IF neuron model, which is closer to real biological neurons and is more computationally powerful. In biological neurons, since the ion exchange inside and outside the cell membrane is not interrupted, if there is only one input stimulus, the potential will leak spontaneously, gradually drop below the resting potential $V_{reset}$, and then rise again to $V_{reset}$. The IF neuron model mentioned earlier is generally considered to fall directly back to the resting state without the leakage process. The subthreshold neural dynamics of the LIF model are described as follows [34]:

(4) $$\begin{array}{c} \tau \frac{dV(t)}{dt}=-\left(V\left(t\right)-V_{reset}\right)+R_{m}I\left(t\right) \end{array}$$

where τ is the time constant, controlling the temporal summation of the synaptic potential. It is equal to the product of the resistance, $R_{m}$, and the capacitance, *C*, of the neuron. The time constant helps determine the time course of the synaptic potential and thereby controls temporal summation, the process by which consecutive synaptic potentials at the same site are added together in the postsynaptic cell. Neurons with a larger membrane time constant have a greater capacity for temporal summation than neurons with a shorter time constant. As a result, the longer the time constant, the greater the likelihood of two consecutive inputs from an excitatory presynaptic neuron summating to bring the cell membrane to its threshold for an action potential.

Again, the discretized updating equation of the LIF neuron is utilized here:

(5) $$\begin{array}{ccc} V(t) & = & \left(1-\frac{1}{\tau }\right)V\left(t-1\right)+\frac{1}{\tau }V_{rest}+X\left(t\right) \end{array}$$

Note that when a spike is fired in a previous time step, i.e., S(V(t−1))=1, the membrane potential starts to accumulate from scratch: $V\left(t\right)=V_{rest}+X\left(t\right)$. Instead, when a spike is not fired in the previous time step, the membrane potential continues to accumulate via a leaky process from the previous step, as $V\left(t\right)=\left(1-\frac{1}{\tau }\right)V\left(t-1\right)+\frac{1}{\tau }V_{rest}+X\left(t\right)$. The membrane potential continues to accumulate from the previous state until it reaches the firing threshold. When the membrane potential at the current time, $V_{t}$, is greater than the threshold value, $V_{th}$, the neuron ejects an output spike. The release of the spike depletes the charge previously accumulated by the neuron, so there is a momentary decrease in the membrane potential, which eventually returns to the resting potential, $V_{reset}$.

### 2.2. The Proposed Network Architecture of an SNN

**Figure 1:**
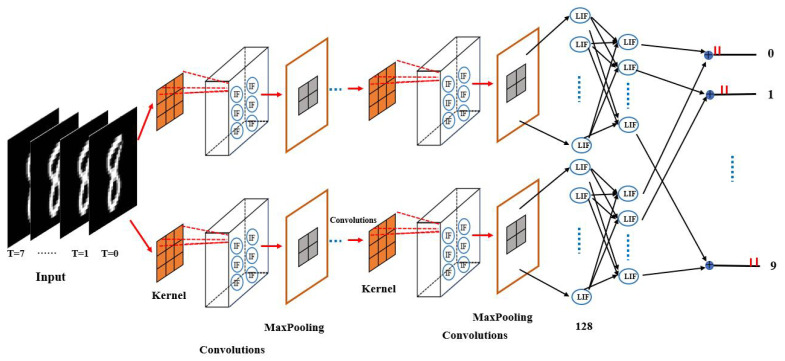
The architecture of the self-adaptive input-encoding spiking neural network with a parallel structure. It has two branches, with each branch containing multiple convolutional layers composed of integrate-and-fire neurons and max pooling layers, a fully connected layer composed of leaky integrate-and-fire neurons, and an output layer also designed by leaky integrate and fire neurons. The output layers of each branch are averaged to form the final output of the network.

The architecture of the proposed self-adaptive coding spiking neural network (SCSNN) with a parallel structure is delineated in Figure 1. The proposed network contains two structurally identical branches with different parameter initialization. The two branches accept the same input information and then process it separately. The processed information of the two branches is finally converged (by way of averaging) to form the final output of the network. The two subnetworks are not completely independent. The converged final outputs cast dependence of one sub-network on another through the backpropagation learning procedure.

Each subnetwork consists of multiple convolutional layers composed of integrate-and-fire (IF) neurons, followed by downsampling maximum pooling layers, a fully connected layer composed of leaky integrate-and-fire (LIF) neurons, and a final output layer also composed of LIF neurons. The IF neuron model is used in the convolutional layers, as it is very computationally efficient. The integration of IF neurons and convolution operations enables the network to encode the input signal (e.g., image) into spikes automatically, avoiding the information loss caused by the separated preprocessing step of Possion encoding.

**Figure 2:**
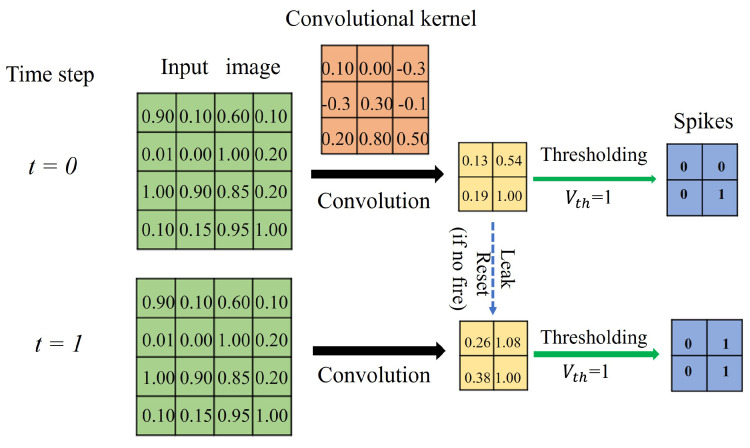
An illustration of the computing process of the input-encoding layer.

The first convolutional layer functions as a sensory neuron. It extracts information from the real-valued input (e.g., image) and encodes it into discrete spikes. It admits real-valued inputs via the convolutional kernel and transforms the information into spikes via the integrate-and-fire model (see Figure 2). The IF neuron model used within the convolutional layer makes the whole network temporally connected in an inter-layer fashion, mimicking the short-term memory capability of biological neurons. At each time step, the raw data are directly input to the network for convolution with the kernel. The convolutional kernel converts the input within the receptive field into the membrane potential of the IF neuron. If the membrane potential at the current time step exceeds the firing threshold, the IF neuron evokes a spike and falls to the resting state; otherwise, the membrane potential leaks into the next time steps for further accumulation. Consequently, the first convolution layer transduces the analog input into binary code, indicating whether a spike is issued.

Following each convolutional layer is a downsampling maximum pooling layer. The maximum pooling layer is used to reduce and optimize the output of the convolutional layer. A convolutional layer and a maximum pooling layer form a module. Multiple convolutional and pooling modules can be added to the first convolutional and pooling module to further extract and optimize the features of the input signal according to the task. The middle convolutional layer is slightly different from the first convolutional layer. It takes discrete spikes encoded by the first convolutional and pooling module for further information processing and transmission.

A fully connected layer composed of LIF neurons is appended to the last convolutional and pooling module. The LIF neuron is utilized here, as it is much more computationally powerful. The fully connected layer combines the features extracted by previous layers to form the output of each subnetwork. The output of each subnetwork.

The output layer of each subnetwork is also composed of LIF neurons. The outputs of each subnetwork are allowed to accumulate over time between the layers to accomplish a specific classification task.

In the output layer of each subnetwork, the weighted spikes accumulate in the membrane potential while decaying with time. At the last time step, the accumulated membrane potential is divided by the total number of time steps, *T*, to quantify the output distribution of the *n*-th subnetwork:

(6) $$\begin{array}{c} o_{n}=\frac{V_{n}^{L}\left(t\right)}{T} \end{array}$$

where *T* denotes the simulation duration and $V_{n}^{L}\left(t\right)$ denotes the membrane potential of the neurons at the last layer (*L*) of the *n*-th subnetwork. The outputs of the subnetworks are combined to form the final output:

(7) $$\begin{array}{c} o=\frac{1}{N}\sum_{n=1}^{N}o_{n} \end{array}$$

where *N* is the number of subnetworks (here, N=2). The class corresponding to the neuron with the largest quantized output value in Equation (7) is used as the label of the input.

Through network architecture design, the data input encoding is incorporated. The network can then accept an analog input and process the information by using discrete action potentials. By using the design of including two branches, redundancy is included so that the robustness of the network is improved, hopefully obtaining improved performance.

### 2.3. Training the Network

The network presented in this study was trained by a surrogate gradient descent backpropagation learning algorithm [35] with our carefully selected substitution gradient of the spiking activity function. The training process contains the forward pass and the backward pass. An input image, *I*, to the network is duplicated *T* times, denoted by $I^{1}\left(t\right)$. The category of the image is encoded by the one-hot vector y. For instance, digit 2 in the MNIST dataset is encoded by the vector (0,0,1,0,0,0,0,0,0,0). For the *n*-th branch of the whole network, the forward pass involves propagating the inputs $I_{n}^{1}\left(t\right)$ in the forward direction to calculate the activations of neurons for each layer *i* up to the output layer *L*. The output from the previous layer of the *n*-th branch $I_{n}^{i-1}\left(t\right)$ is combined to form the input of the current layer $X_{n}^{i}\left(t\right)$:

(8) $$\begin{array}{ccc} X_{n}^{i}\left(t\right) & = & W_{n}^{i-1}I_{n}^{i-1}\left(t\right) \end{array}$$

The input $X_{n}^{i}\left(t\right)$ is then integrated into the membrane potential by the IF model (except the last two layers) via the following equation:

(9) $$\begin{array}{ccc} V_{n}^{i}\left(t\right) & = & V_{n}^{i}\left(t-1\right)+X_{n}^{i}\left(t\right) \end{array}$$

or the LIF model (for the last two layers) via the following equation:

(10) $$\begin{array}{ccc} V_{n}^{i}\left(t\right) & = & \left(1-\frac{1}{\tau_{n}^{i}}\right)V_{n}^{i}\left(t-1\right)+\frac{1}{\tau_{n}^{i}}V_{rest}^{n}+X_{n}^{i}\left(t\right) \end{array}$$

where the neurons at layer *i* share the time constant, $\tau_{n}^{i}$, computed by $\tau_{n}^{i}=1+\exp\left(-a_{n}^{i}\right)$. Here, $a_{n}^{i}$ is a learnable parameter. The membrane potential $V_{n}^{i}\left(t\right)$ converts into spikes if exceeding the firing threshold $V_{th}^{n}$:

(11) $$\begin{array}{c} I_{n}^{i}\left(t\right)=S\left(V_{n}^{i}\left(t\right)\right) \end{array}$$

Note the output spikes at the current layer, $I_{n}^{i}\left(t\right)$, of the *n*-th branch are the input spikes of the next layer of this branch. The output of the *n*-th branch of the network is the averaged spikes emitted by the last layer:

(12) $$\begin{array}{c} o_{n}=\frac{1}{T}\sum_{t=0}^{T-1}I_{n}^{L}\left(t\right) \end{array}$$

The outputs of the whole network are the averaged outputs of the two branches:

(13) $$\begin{array}{c} o=\frac{1}{N}\sum_{n=1}^{N}o_{n} \end{array}$$

The network is trained in a way that minimizes the loss function defined by the $L_{2}$ norm of the difference between the true label vector and the output vector of the network:

(14) $$\begin{array}{c} E=\frac{1}{2}{\left\|o-y\right\|}_{2}^{2} \end{array}$$

The gradient of the loss function *E* with respect to the network output o equals the final output error:

(15) $$\begin{array}{c} \frac{\partial E}{\partial o}=\frac{\partial }{\partial o}\frac{1}{2}{\left\|o-y\right\|}_{2}^{2}=o-y=e \end{array}$$

The output error, e, is then backpropagated to the previous layers through the gradient of the loss function with respect to the parameter $W_{n}^{i-1}$ of each layer for the *n*-th branch, which is calculated via the chain rule as follows:

(16) $$\begin{array}{c} \frac{\partial E}{\partial W_{n}^{i-1}}=\frac{\partial E}{\partial o}\frac{\partial o}{\partial o_{n}}\frac{\partial o}{\partial W^{i-1}}\\ =\frac{e}{TN}\sum_{i=0}^{T-1}\left\{\left(\frac{\partial I_{n}^{i}\left(t\right)}{\partial V_{n}^{i}\left(t\right)}+\frac{\partial I_{n}^{i}\left(t+1\right)}{\partial V_{n}^{i}\left(t+1\right)}\frac{\partial V_{n}^{i}\left(t+1\right)}{\partial V_{n}^{i}\left(t\right)}\right)\right.\\ \left.\cdot \frac{\partial V_{n}^{i}\left(t\right)}{\partial X_{n}^{i}\left(t\right)}I_{n}^{i-1}\left(t\right)\right\} \end{array}$$

Similarly, the gradient of the loss function with respect to the parameter $a_{n}^{i}$ is calculated as follows:

(17) $$\begin{array}{c} \frac{\partial E}{\partial a_{n}^{i}}=\frac{e}{T}\sum_{i=0}^{T-1}\left\{\left(\frac{\partial I_{n}^{i}\left(t\right)}{\partial V_{n}^{i}\left(t\right)}+\frac{\partial I_{n}^{i}\left(t+1\right)}{\partial V_{n}^{i}\left(t+1\right)}\frac{\partial V_{n}^{i}\left(t+1\right)}{\partial V_{n}^{i}\left(t\right)}\right)\right.\\ \left.\cdot \frac{\partial V_{n}^{i}\left(t\right)}{\partial a_{n}^{i}}\right\} \end{array}$$

However, the gradient $\frac{\partial I_{n}^{i}\left(t\right)}{\partial V_{n}^{i}\left(t\right)}$ cannot be computed as the neuron activation function S(·) given by Equation (3) is not differentiable. Thus, we cannot train our model directly by using the backpropagation gradient descent algorithm. A gradient substitution is used to solve this problem. In this paper, the derivative of the arctangent function or sigmoid function was chosen to replace the gradient of the neuron activation function S(·) given by Equation (3). The arctangent and sigmoid functions are defined as follows:

(18) $$\begin{array}{cc} f_{1}\left(x\right) & =\frac{1}{\pi }arctan\left(\frac{\pi }{2}\alpha x\right)+\frac{1}{2} \end{array}$$

(19) $$\begin{array}{cc} f_{2}\left(x\right) & =\frac{1}{1+e^{-\alpha x}} \end{array}$$

with derivatives given by

(20) $$\begin{array}{cc} {f_{1}}^{\prime }\left(x\right) & =\frac{\alpha }{2+\frac{\pi^{2}}{2}\alpha^{2}x^{2}} \end{array}$$

(21) $$\begin{array}{cc} {f_{2}}^{\prime }\left(x\right) & =\frac{\alpha e^{-\alpha x}}{1+2e^{-\alpha x}+e^{-2\alpha x}} \end{array}$$

**Figure 3:**
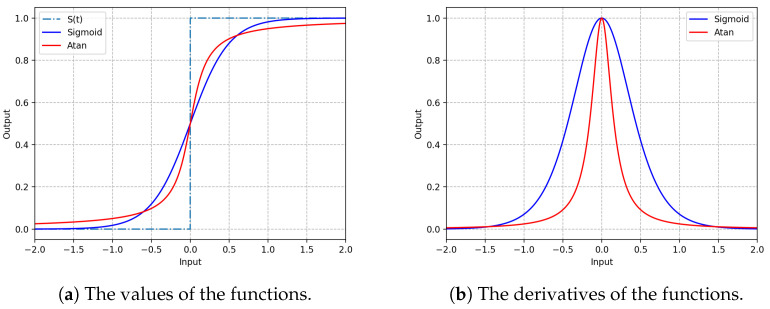
The values and derivatives of the sigmoid and arctangent functions that approximate the nondifferentiable spike-emitting set function.

Here, α is a user-specified parameter that controls the degree of smoothness of the function. A larger α leads to a better approximation of the step function. However, a larger α can also result in a higher chance that the gradient of the network explodes when the input of this function is close to 0 and vanishes when far away from 0. Figure 3 gives an illustration of the arctangent and sigmoid functions with α=4.0. Figure 3a shows that the continuous arctangent function or sigmoid function can well approximate this spike-firing process. Compared with the sigmoid function, the arctangent function is closer to the step function when the difference between the membrane potential $V_{t}$ and the threshold $V_{th}$ is small. Its gradient is steeper than that of the sigmoid function, as shown in Figure 3b.

## 3. Results

In this section, we present the experimental results. Three popular publicly available image datasets, namely MNIST, Fashion-MNIST, and CIFAR10, were used to verify the effectiveness of the proposed networks.

The proposed networks were implemented in Python under the PyTorch framework. The weights of the network were randomly initialized. To prevent overfitting, a dropout strategy was used, which randomly set 40% of the nodes in each fully connected layer to 0. Moreover, to prevent gradient disappearance and explosion, the batch normalization (BN) method was used, which normalizes the mean and variance of the outputs of each layer during the network training process.

For the MNIST dataset, which contains grayscale images of handwritten digits from 0 to 9, the original 28×28 pixel images were used. No preprocessing was applied. The standard training/test split was used, where 60,000 images (around 6000 images per digit) were used for training, and 10,000 images (about 1000 images per digit) were used for testing.

For the Fashion-MNIST dataset, which contains grayscale images of clothing belonging to 10 types, the original 28×28-pixel images without any preprocessing were used. The standard training/testing split was used, where 60,000 images (around 6000 images per type) were used for training, and 10,000 (about 10,000 images per type) were used for testing.

For the CIFAR10 dataset, which contains color images of objects belonging to 10 categories, the normalized 32×32 pixel color images were used. The color images were preprocessed into R, G, and B channels, with each channel normalized to a zero mean and unit variance.

The standard training/testing split was used, where 50,000 images (about 5000 images per category) were used for training, and 10,000 images (around 1000 images per category) were used for testing.

### 3.1. Input-Encoding Performance

**Figure 4:**
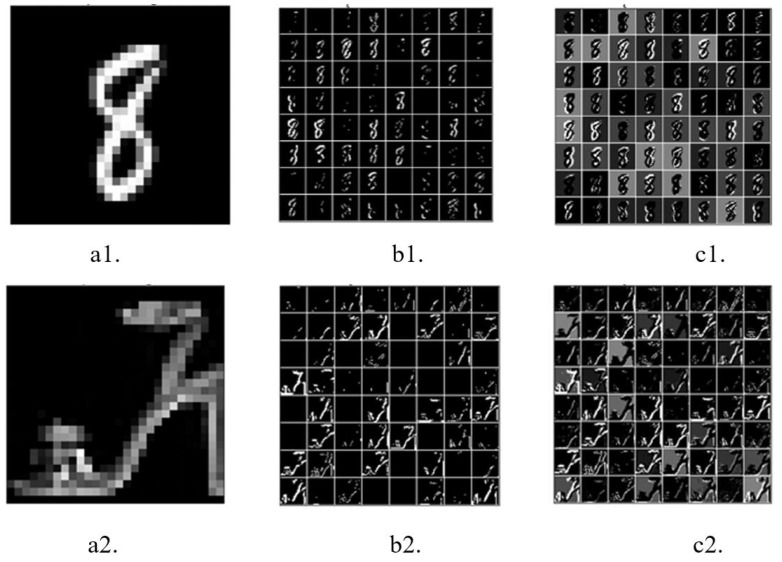
The performance of the input-encoding layer. (**a1**,**a2**) show the raw images from the MNIST and Fashion MNIST datasets, respectively. (**b1**,**b2**) present the initial feature maps of the images from the MNIST and Fashion MNIST datasets, respectively (simulation time T=0). (**c1**,**c2**) illustrate the feature maps of the images from the MNIST and Fashion MNIST datasets, respectively, at simulation time T=7.

The raw versions of Figure 4(a1,a2) were directly input into the network. The first convolutional layer encoded the raw images into feature maps containing spikes (spike map). At the beginning of the simulation (T=0), the corresponding output feature maps of the first convolutional layer only partially encoded the original images (see Figure 4(b1,b2), respectively). As the simulation continued, the contours of the objects in the original images became clearer in the output feature maps (see Figure 4(c1,c2)), with a simulation time of T=7. These results indicate that the first convolutional layer can encode the raw image into spikes well.

**Figure 5:**
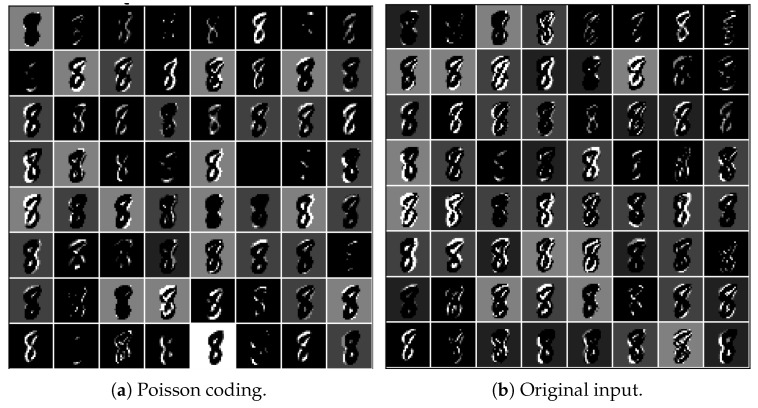
Encoding performance comparison of the proposed network between the original input and the spike input obtained using Poisson coding.

Due to the flexibility of the presented architecture, the network can also accept spike inputs encoded by Poisson coding, just as classic spiking neural networks do. By encoding the original image using Poisson coding and inputting the encoded spikes into the presented network, we obtained the output of the first convolutional layer, as given in Figure 5a. Compared with Figure 5b, which was generated by inputting the original image, the contours of the digit “8” are more blurred. Some features even vanished. This indicates that the prepocessing of images using Poisson coding may result in loss of information. Instead, the network that can accept raw images takes in the full information. Therefore, it is more advantageous in that it can only admit spikes in terms of information processing.

### 3.2. Effects of Different Surrogate Gradients on Model Performance

**Figure 6:**
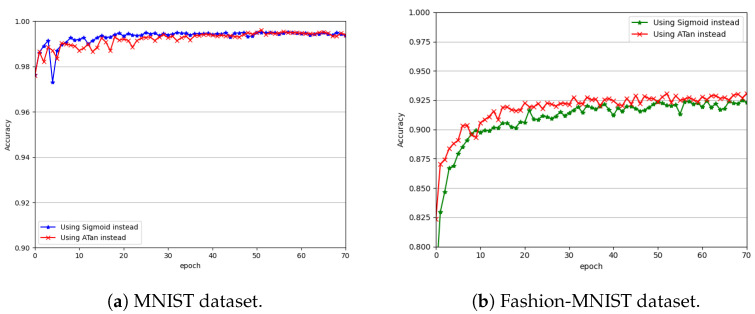
Performance convergence curves of the arctangent surrogate gradient compared with that of the sigmoid surrogate gradient.

The gradient of the sigmoid function or arctan function was used as the surrogate gradient for the nondifferentiable step function, respectively. Figure 6a compares the classification performance of the presented network with the two surrogate gradients on the MNIST dataset for simulation times of T=8 and α=4.0. Figure 6b does so using the Fashion-MNIST dataset.

From Figure 6a,b, we can see that the network using either of the surrogate gradients achieved good convergence. After being trained only for 30 epochs, the performance of the network using either of the surrogate gradients started to converge on both datasets. However, compared with the sigmoid function, the network using the surrogate gradient of the arctangent function converged to provide slightly better performance. The network using the surrogate gradient of the arctangent function also showed better stability on both datasets. One possible reason is that the arctangent function approximates the step function better (see Figure 3a) and, thus, is more conducive to the modeling of the nonlinear characteristics of the network. Moreover, compared with that of the sigmoid function, the gradient of the arctangent function is faster to compute and, thus, is more favorable to the training of the network. Therefore, in this paper, we recommend the arctangent surrogate gradient, and we performed all the subsequent experiments using this surrogate gradient.

### 3.3. Impact of Simulation Time on Network Performance

Spiking neural networks use spike events (consisting of 0 s or 1 s) to characterize and transmit information. Neurons accumulate information obtained from upstream neurons in time to trigger spike events to pass information to downstream neurons. Therefore, the number of time steps that are used to accumulate information has a great impact on the performance of the network. If the number of time steps were too small, the accumulated sum of the IF neurons or LIF neurons would not be enough to generate output spikes. Thus, SNN could not receive enough information for training or inference, resulting in bad performance. In contrast, a large number of time steps would reduce the randomness of the network and eliminate noise and inaccuracy, but this would be at the cost of high latency and high computational power consumption. Therefore, a suitable number of time steps needs to be considered to balance the trade-off between the performance of the network and the effort to train the network.

**Figure 7:**
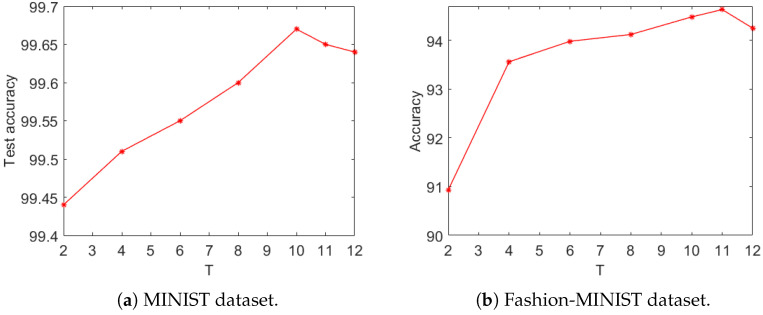
The impact of the number of simulation time steps on the performance of the proposed network.

As the presented network used a self-adaptive encoding strategy, the required number of time steps was substantially smaller than that used in Poisson encoding. A conventional SNN that used Poisson encoding required time steps in the hundreds, while the presented network required time steps only in units or tens. Thus, we only experimented using 2 to 12 time steps. The impacts of different numbers of time steps on the proposed network are demonstrated in Figure 7.

Figure 7a shows the accuracy curves as functions of the number of time steps on the MNIST dataset. Generally speaking, with increasing time steps, *T*, the test accuracy increased. However, the accuracy became saturated at T=10, and as *T* increased further, the test accuracy started to decrease. A similar trend was observed on the Fashion-MNIST dataset (see Figure 7b).

### 3.4. Effect of Parallel Construction

To verify the robustness of the parallel SCSNN network, we compared the parallel network with the single-branched network and also performed lesion experiments. In the lesion experiments, we fully trained the parallel network and then deliberately disabled one branch of the fully trained parallel SCSNN network and only tested the remaining untouched branch. Table 1 demonstrates the corresponding results on the MNIST dataset. Disabling one branch of the fully trained parallel SCSNN network made the test accuracy of the network converge slightly worse. The fully trained parallel SCSNN network with the left branch disabled obtained an accuracy of 99.58%, worse than the fully trained parallel SCSNN network without any lesions (99.72%). It also performed slightly worse than the single SCSNN network, with a test accuracy of 99.62%.

This corresponds with our understanding of the vision system. People who grew up with two fully functioning eyes showed worse sight when they closed one of their eyes than when opening two eyes; they also showed slightly worse sight than people who grew up with only one fully functioning eye.

**Figure 8:**
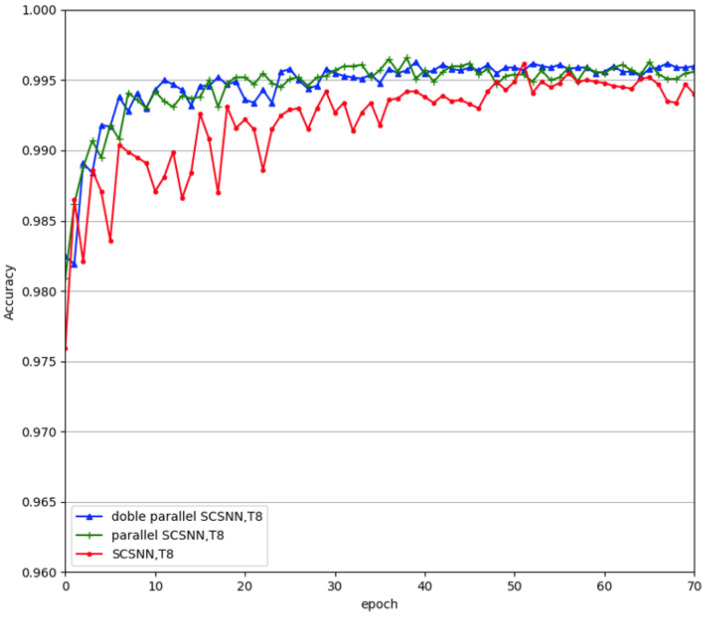
Performance comparison of the double parallel SCSNN model.

Our experiments have shown that parallel construction increased the recognition accuracy of the network, as well as the stability and robustness. We further explored whether an increased degree of redundancy would result in a further increase in recognition accuracy and stability. To this end, we doubled the parallel construction to make four subnetworks work in parallel. The experimental results are given in Figure 8. From the figure, we can see that increasing the number of subnetworks further, from two to four, did not increase the performance of the network further. From the neurological point of view, redundancy, indeed, increases robustness in the nervous system, but it also increases energy consumption. Thus, from an evolutionary point of view, the best strategy is just to maintain a certain degree of redundancy. From a computational point of view, the performance of the network will eventually saturate with increasing complexity (e.g., expansion of width in this study) for a given dataset. Further increasing the complexity of the network may jeopardize its generalization performance due to the phenomenon of overfitting. Thus, we cannot arbitrarily increase the branches in the parallel construction. Moreover, with the expansion of the width of the network, the training time increased exponentially. Consequently, we recommend parallel construction using two subnetworks in this study.

### 3.5. Comparison with State-of-the-Art SNN Design

We finally compared the suggested SNN architecture with current state-of-the-art SNN designs on the MNIST and Fashion-MNIST datasets, as well as a more complicated dataset named CIFAR10. The number of time steps over which the membrane potential of the spiking neuron accumulates, the learning rate, the batch size used in the batch normalization, the training epochs, and the surrogate gradient were manually selected based on the accuracy of a small validation set split from the training set. The reported accuracy was the test accuracy of the pre-split test set. The leaky item of the leaky integrate-and-fire model was set to 1. The utilized hyperparameters in this paper are given in Table 2. The results using the three datasets are listed in Table 3, Table 4 and Table 5, respectively. The recognition accuracy of the compared methods was taken from their corresponding publications.

The compared methods include the following:

- *Spiking CNN* [36] was designed as a competitive recurrent network by adding winner-take-all circuits, where only one neuron in a group of neurons is allowed to fire via the lateral inhibitory connections among the neurons within the group. The network was trained by using a gradient descent algorithm, where the nondifferentiable problem of spike events was solved by adding a low-pass filtered pulse signal to the membrane potential and treating abrupt changes in membrane potential as noise during error backpropagation.
- *Spike layer error reassignment (SLAYER)* [37] was designed using a temporal credit assignment policy for backpropagating errors to preceding layers in order to overcome the problem of the nondifferentiability of the spike function. This enabled SLAYER to simultaneously learn both synaptic weights and axon delays.
- *Spatio-temporal backpropagation (STBP)* [14] was used to train the SNN by combining the layer-by-layer spatial domain (SD) and the timing-dependent temporal domain (TD). The SNN constitutes iterative LIF models that are appropriate for gradient descent training. The nondifferentiable issue of spike activity was addressed by using derivative approximation.
- *Hybrid macro/micro level backpropagation (HM2-BP)* [25] was used to train the multi-layer SNN by capturing the temporal effects using spike-train level post-synaptic potential at the microscopic level and defining the rate-coded error at the macroscopic level, which was computed and backpropagated across both macroscopic and microscopic levels.
- *Spike-train level recurrent SNNs backpropagation (ST-RSBP)* [38] was used to train the deep recurrent spiking neural network (RSNN) by directly computing the gradient of a rate-coded loss function with respect to the learnable parameters without incurring the approximations resulting from altering and smoothing the underlying spiking behaviors.
- *Spiking neural networks with lateral interactions (LISNN)* [20] utilized LIF models for which membrane potential updation was used to take the lateral interactions between neighbor neurons into consideration. The network was trained by using backpropagation gradient descent. The nondifferentiable problem of the neuron activation function was solved by appointing a pseudo derivative to it.
- *Error-driven local representation alignment (LRA-E)* [39] was used to train SNNs by minimizing the local loss between the representation and the predicted target for each layer (motivated by predictive coding). The target of each layer was predicted as the nonlinear projection of the displacement obtained from the previous layer. LRA-E moved the network parameters in a direction that was not too far away from the direction pointed to by backpropagation, but it was more biologically plausible than backpropagation.
- DL-BP uses ReLU as an activation function for the hidden layer in a deep neural network [40]. This is achieved by activating the penultimate layer in the spiking neural network and then using it to learn the weight parameters of the ReLU classification layer by using backpropagation.

As shown in Table 3, on the MNIST dataset, the presented parallel SCSNN network obtained an accuracy of 99.72%, outperforming other existing SNN designs in the literature at 0.1% better than the best existing result delivered by spike-train level recurrent spiking neural networks backpropagation (ST-RSBP) [38]. On the Fashion-MNIST dataset, the presented parallel SCSNN network reached an accuracy of 94.63%, being 2.7% better than the existing best SNN design called lateral iteration spiking neural network (LISNN) [20] (see Table 4). On the more complicated CIFAR10 dataset, the proposed parallel SCSNN network obtained an accuracy of 91.43%, which is comparable to existing deep spiking neural networks (see Table 5). Note that the compared networks have seven to nine valid layers, but our network only has five valid layers. The results indicate the effectiveness of parallel SCSNN construction.

Note that for the MNIST and Fashion-MNIST datasets, we only used four valid layers (in Table 3, Table 4 and Table 5, “P” means the pooling layer and is not included when counting the number of valid layers). This first convolutional layer is used to encode the input into spike trains, mimicking the information-processing process of the periphery receptors. The middle two convolutional layers perform feature extraction. The final fully connected layer combines the features extracted by the previous layers to form the output of the neural network. We can always reduce the number of valid layers to three by using only one valid layer for feature extraction. However, the generalization performance will be reduced as well. We can also increase the number of valid layers to more than four by using more than two valid layers for feature extraction. This will result in more computational time and may also lead to degenerated performance due to the overfitting problem. To reach a balance, we utilized four valid layers. However, for the more complicated CIFAR10 dataset, we tried to use four valid layers; it did not achieve desirable generalization performance. Thus, we used five valid layers, which showed competitive performance compared to the existing SNNs. Still, we used less valid layers than the compared SNN designs.

Our proposed SNN integrated the process of encoding the analog input into spikes in the whole architecture. The experimental results revealed that the integration of the input spike-coding scheme can fully propagate the information within the input with a shorter simulation time than that using a separate input spike-coding scheme. The inclusion of a parallel structure further improved the robustness and the generalization performance when compared to the network with a single branch. In addition to superior generalization performance, our proposed SNN architecture is more biologically plausible. It successfully imitated the integrated information-processing scheme of the biological neuron system, starting from the periphery receptors to the central nervous system. Our SNN architecture design provides an alternative, preferable design for an SNN and simultaneously suggests the advantages of using the biological neuron system.

## 4. Discussion and Conclusions

The human brain is an exquisite “computing machine”, resulting from evolution over millions of years. The working mechanism behind the human brain has inspired many mathematical methods and algorithms. Among them, the artificial neural network (ANN) is very successful. However, ANNs process and transmit information using the firing rates of spikes in the brain, which is highly rough and condensed, ignoring the temporal information between the spikes that may also convey important information. To take advantage of this temporal information and come closer to how the brain works, spiking neural networks (SNNs) that process and transmit information using spike trains are emerging.

However, most of the existing SNNs require a separate spike-encoding preprocessing step to convert the real-valued inputs into discrete spikes first and then input the spikes to the network for further processing [21]. The spike-encoding process is dissected from the spiking neural network and is not trained together with the subsequent network. Nevertheless, the biological neuron system does not perform a separate preprocessing step. Instead, the neural pathway directly admits the input from the environment via the sensory receptors to convert analog stimuli into corresponding discrete spikes, subsequently relaying information to the central nervous system for further processing. The sensory receptors are not independent of the central neural system but are modulated by the central neural system. Moreover, the study of the biological brain has revealed that the nervous system may have multiple circuits to perceive the same stimulus [27], including redundancy in terms of improving the robustness of the nervous system [29,30].

Inspired by these advantageous aspects of the biological neural system, we suggest a self-adaptive encoding spike neural network with parallel architecture. The proposed network integrates the input-encoding process into the spiking neural network architecture via convolutional operations such that the network can accept the real-valued input and automatically transform it into spikes for further processing. Meanwhile, the proposed network contains two identical parallel branches inspired by the way the biological nervous system processes information in both serial and parallel. The proposed network is trained by a surrogate gradient descent algorithm with carefully chosen gradient substitution for the nondifferentiable spike-emitting step function. The experiments on multiple image recognition tasks showed the proposed SNN could obtain comparable or even better performance than existing SNNs, indicating the advantages of the integration of spiking encoding and parallel architecture.

In addition to competitive recognition performance, the proposed models have lower simulation time, fewer training iterations, and faster convergence compared to existing SNN models that use Poisson coding for input spike encoding. However, the proposed network only roughly exploits part of the brain’s mechanisms. In our future work, we will consider including more brain mechanisms in our network design, such as lateral interactions and recurrent connections. A more biologically plausible network learning algorithm than the surrogate gradient descent algorithm may also be considered. This work only applied the proposed SNN to three image classification tasks. In our future work, we will consider adapting our proposed SNN to deal with more complicated tasks, such as speech recognition and time series analysis.

## Figures and Tables

**Table 1:** Test accuracy comparison among parallel SCSNN, lessioned parallel SCSNN, and SCSNN on the MINIST dataset.

Methods	Accuracy(%)
Parallel SCSNN	99.72
Parallel SCSNN with left branch lesion	99.58
SCSNN	99.62

**Table 2:** Hyperparameters of parallel SCSNN.

Hyperparameters	MNIST	Fashion-MNIST	CIFAR10
No. of time steps	10	11	10
Learning rate	0.0002	0.0005	0.0001
Batch size	128	128	32
Training epochs	60	120	140
Leaky item	1	1	1
Surrogate gradient	Atan	Atan	Sigmoid

**Table 3:** Performance of the proposed networks compared with existing SNN methods on MNIST.

Methods	Hidden Layers	Accuracy %
Spiking CNN [36]	20C5−P2−50C5−P2−200FC	99.31
SLAYER [37]	12C5−P2−64−P2	99.41
STBP [14]	12C5−P2−40C5−P2−300FC	99.42
HM-2BP [25]	15C5−P2−40C5−P2−300FC	99.49
ST-RSBP [38]	15C5−P2−40C5−P2−300FC	99.62
LISNN [20]	32C3−P2−32C3−P2−128FC	99.50
Spiking BP [34]	6C5−P2−16C5−P2−120FC−84FC	99.59
Parallel SCSNN	32C3−P2−64C3−P2−256C3−P2−560FC	99.72

**Table 4:** Performance of the proposed networks compared with existing SNN methods on Fashion-MNIST.

Methods	Hidden Layers	Accuracy %
LRA-E [39]	5×256	87.69
HM-2BP [25]	400−40	88.99
DL-BP [40]	3×512	89.06
ST-RSBP [38]	400−R400	90.13
LISNN [20]	32C3−P2−32C3−P2−128FC	92.07
Parallel SCSNN	32C3−P2−128C3−P2−256C3−P2−560FC	94.63

**Table 5:** Performance of the proposed networks compared with existing SNN methods on CIFAR10.

Methods	Time-Steps	Number of Layers	Accuracy %
ANN-SNN [41]	2500	ResNet9	91.55
SNN [17]	390	128C3−256C3−P2−512C3−P2−1024C3−512C3−1024FC−512FC	90.53
Spiking BP [34]	100	ResNet9	90.35
Spiking BP [34]	100	VGG9	90.45
DIET-SNN [42]	10	VGG6	90.05
Parallel SCSNN	10	128C3−P2−256C3−P2−512C3−P2−512C3−1024FC	91.43

## Data Availability

The data presented in this study are available upon request from the corresponding author.

## Abbreviations

The following abbreviations are used in this manuscript:

SNN	Spiking neural network
ANN	Artificial neural network
STDP	Spike-timing-dependent plasticity
CNN	Convolutional neural network
DOG	Difference-of-Gaussian
SCSNN	Self-adaptive coding spiking neural network
BP	Propagation
IF	Integrate-and-fire
LIF	Leaky integrate-and-fire
